# RELIGIOSITY AND SPIRITUALITY DEVELOPMENT ACROSS MID- TO LATER LIFE: AN ACCELERATED LONGITUDINAL DESIGN

**DOI:** 10.1093/geroni/igad104.0075

**Published:** 2023-12-21

**Authors:** Niccole Nelson, Raquael Joiner, Brandy Martinez, Cindy Bergeman

**Affiliations:** Colorado State University, Fort Collins, Colorado, United States; University of Southern California, Los Angeles, California, United States; Durham VAHCS, Durham, North Carolina, United States; University of Notre Dame, Notre Dame, Indiana, United States

## Abstract

It is widely accepted that religiosity and spirituality (R/S) increase with age, although the multidimensional nature of R/S complicates this conclusion. Specifically, religiosity entails engagement in communal and independent religious practices, whereas spirituality refers to a sense of communion with divine being(s). Furthermore, religious and/or spiritual individuals may use their faith to carry them through hard times, and thus engage in positive and/or negative religious/spiritual coping. Critically, these R/S facets may follow distinct developmental trajectories across adulthood, and the nature of these trajectories remain unclear. Therefore, in the present study, we followed an accelerated longitudinal design, modeling developmental trends in six R/S facets across ages 45-80. Participants included 768 subjects from the Notre Dame Study of Health & Well-being (NDHWB), a 10-year, multi-cohort, longitudinal study of adult development and aging (Wave 1 Age: Mean= 59.24, SD= 8.65). Using two-level, hierarchical linear modeling, we estimated no change, linear change, quadratic change, and cubic change trajectories across ages 45-80 in communal and independent religious practices, spirituality, positive and negative religious/spiritual coping, as well as composite R/S; cohort effects in these change trajectories were also tested. Communal and independent religious practices, spirituality, negative religious/spiritual coping, as well as composite R/S, followed unique, cubic trajectories across mid- to later life. Positive religious/spiritual coping followed a linear trajectory, although cohort effects precluded the interpretation of developmental change in this R/S facet. Individuals appear to engage with their faith in different ways as they age, meaning extant conclusions about age-graded increases in R/S may be oversimplified.

